# Erythrocytes and Vascular Function: Oxygen and Nitric Oxide

**DOI:** 10.3389/fphys.2018.00125

**Published:** 2018-02-22

**Authors:** Christine C. Helms, Mark T. Gladwin, Daniel B. Kim-Shapiro

**Affiliations:** ^1^Physics Department, University of Richmond, Richmond, VA, United States; ^2^Heart, Lung, Blood and Vascular Medicine Institute, University of Pittsburgh, Pittsburgh, PA, United States; ^3^Division of Pulmonary, Allergy, and Critical Care Medicine, Department of Medicine, University of Pittsburgh, Pittsburgh, PA, United States; ^4^Physics Department, Wake Forest University, Winston-Salem, NC, United States; ^5^Translational Science Center, Wake Forest University, Winston-Salem, NC, United States

**Keywords:** erythrocytes, hemoglobin, hemolysis, hypoxic vasodilation, nitric oxide, nitrite

## Abstract

Erythrocytes regulate vascular function through the modulation of oxygen delivery and the scavenging and generation of nitric oxide (NO). First, hemoglobin inside the red blood cell binds oxygen in the lungs and delivers it to tissues throughout the body in an allosterically regulated process, modulated by oxygen, carbon dioxide and proton concentrations. The vasculature responds to low oxygen tensions through vasodilation, further recruiting blood flow and oxygen carrying erythrocytes. Research has shown multiple mechanisms are at play in this classical hypoxic vasodilatory response, with a potential role of red cell derived vasodilatory molecules, such as nitrite derived nitric oxide and red blood cell ATP, considered in the last 20 years. According to these hypotheses, red blood cells release vasodilatory molecules under low oxygen pressures. Candidate molecules released by erythrocytes and responsible for hypoxic vasodilation are nitric oxide, adenosine triphosphate and S-nitrosothiols. Our research group has characterized the biochemistry and physiological effects of the electron and proton transfer reactions from hemoglobin and other ferrous heme globins with nitrite to form NO. In addition to NO generation from nitrite during deoxygenation, hemoglobin has a high affinity for NO. Scavenging of NO by hemoglobin can cause vasoconstriction, which is greatly enhanced by cell free hemoglobin outside of the red cell. Therefore, compartmentalization of hemoglobin inside red blood cells and localization of red blood cells in the blood stream are important for healthy vascular function. Conditions where erythrocyte lysis leads to cell free hemoglobin or where erythrocytes adhere to the endothelium can result in hypertension and vaso constriction. These studies support a model where hemoglobin serves as an oxido-reductase, inhibiting NO and promoting higher vessel tone when oxygenated and reducing nitrite to form NO and vasodilate when deoxygenated.

How erythrocytes modulate vascular tone has been widely studied over the last two decades. The vasodilation of the vasculature under hypoxic conditions has inspired much research ranging from the effect of oxygen partial pressure on smooth muscle cell contractility and endothelial nitric oxide synthase (eNOS) activity to nitrite reduction by hemoglobin (Hb) inside erythrocytes and subsequent production of nitric oxide (Figure 1). Here we review how red blood cells (RBCs) and hemoglobin regulate vascular function and blood flow.

**Figure 1:**
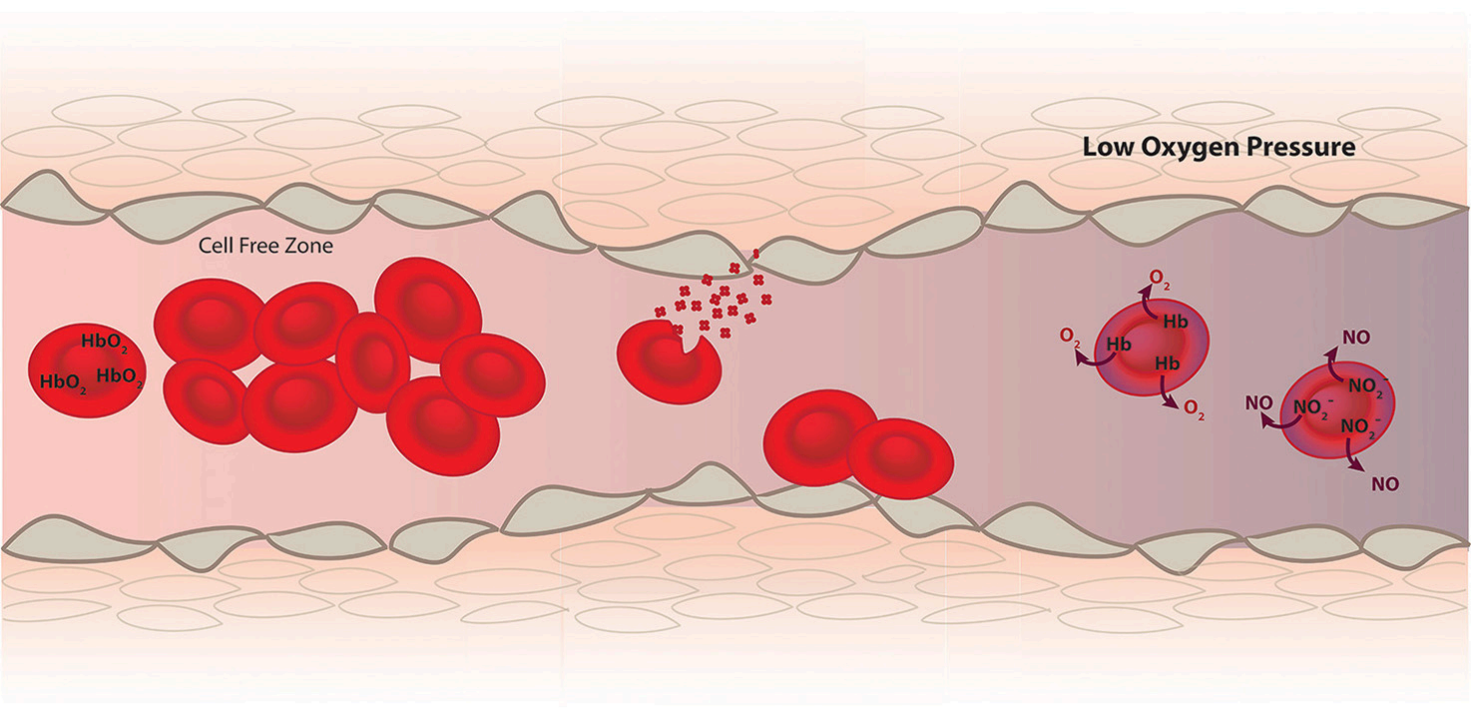
RBCs function as a transporter of oxygen from the lungs to the tissue and help establish hemostasis and vascular function. Since Hb inside RBCs is a very effective scavenger of NO, a vasodilator produced by the endothelium (brown), mechanism such as a cell free zone created by fluid dynamics, RBC membrane and internal diffusion minimize NO scavenging. In diseases where hemolysis and RBC adhesion occur these mechanisms to minimize NO scavenging are compromised and vasoconstriction occurs. However, in addition to scavenging NO data supports a role of deoxygenated RBCs in the production of NO leading to vasodilation under hypoxic conditions.

## Oxygen delivery

Many biochemistry texts provide thorough descriptions of the basic mechanisms of oxygen delivery by hemoglobin. Therefore, we will limit our discussion to a brief introduction. The hemoglobin molecule is a heterotetramer of hemoglobin alpha-beta dimers. Under high oxygen tension, each monomer can bind an O_2_ molecule. As hemoglobin binds oxygen, conformational changes occur to the protein and the binding affinity of the protein for molecular oxygen increases. Thus, binding is cooperative so that binding subsequent molecules of oxygen becomes easier after initial binding. Cooperative binding can be understood by a two-state model: The high affinity R-state and the low affinity T-state (Monod et al., 1965; Eaton et al., 1999). When hemoglobin has one or less oxygen molecules bound it is virtually always in the tense T-state. When it has 3–4 oxygen molecules bound it is virtually always in the R-state. Once 2 or more oxygen bind to a hemoglobin molecule that has no oxygen it will undergo a conformational change to the R-state where its oxygen affinity is now higher and it binds more oxygen. This homotropic regulation leads to a sigmoidal curve of hemoglobin fractional saturation vs. partial pressure of oxygen and allows hemoglobin to function as an efficient transporter of oxygen from the lungs to the tissues.

The complexity of oxygen delivery goes beyond the homotropic regulation. The pH of the blood also effects oxygen binding. The Bohr Effect, reported in the early 1900's, describes how increased acidity which occurs at low oxygen tension leads to protonation and stabilization of the T-state of hemoglobin promoting oxygen release. Additionally, carbon dioxide and 2,3-bisphosphate also act as heterotropic regulators, both decreasing the affinity of Hb for oxygen.

Therefore, RBCs contribute to vascular function through the delivery of oxygen by hemoglobin.

One mechanism through which oxygen tension and therefore RBCs effect vascular function is the contractile force of smooth muscle cells. Chang and Detar reported that a reduction in oxygen pressure led to a reduced contractile tension of helical strips cut from aorta, femoral arteries and small arteries from skeletal muscle (Chang and Detar, 1980). Additionally Taggart et al saw a decrease in smooth muscle force in hypoxia indicating smooth muscle response to oxygen may be independent of factors from the endothelium (Taggart and Wray, 1998). Potential mechanics of smooth muscle response to oxygen tension are increased ATP-sensitive K^+^ efflux or decreased voltage-sensitive Ca^+2^ influx (Franco-Obregón et al., 1995; Taggart and Wray, 1998). These effects have been recently reviewed (Jackson, 2016).

Alternatively, low oxygen tension effects the endothelial production of nitric oxide (NO) by eNOS, which requires oxygen as a substrate along with L-arginine (Palmer and Moncada, 1989; Kwon et al., 1990). Many researchers have shown diminished relaxation by endothelial cells under low oxygen tension (Furchgott and Zawadzki, 1980; De Mey and Vanhoutte, 1983; Lieberthal et al., 1989). Specifically, the apparent K_m_ of eNOS for O_2_ is 4 μM and NOS activity begins to slow down once oxygen tensions starts falling below 1% (7.6 torr or 10 μM) (Abu-Soud et al., 2000; van Faassen et al., 2009). In summary, through the management of oxygen delivery RBCs effect eNOS function and smooth muscle contractility.

## Compartmentalization and localization of hemoglobin

In addition to oxygen delivery, hemoglobin affects vascular function as a strong scavenger of NO. Furchgott and coworkers discovered that there is an agent produced in the endothelium that relaxes blood vessels, the endothelium relaxation factor (EDRF) (Furchgott and Zawadzki, 1980). The EDRF was then identified as NO (Ignarro et al., 1987; Palmer et al., 1987) that acts via activation of soluble guanylyl cyclase (Arnold et al., 1977). Endothelial nitric oxide synthase located on endothelial cell membranes produces NO that binds to soluble guanylyl cyclase in smooth muscles cells triggering a cell-signaling cascade that results in vasodilation. Chu et al. demonstrated the physiological regulation of blood flow and vasomotion by endogenously produced NO in awake dogs (Chu et al., 1991) and Quyyumi et al. showed atherosclerosis risk factors altered the response of coronary arteries to diminished NO production (Quyyumi et al., 1995). Additionally, NO has the anti-thrombotic effect of diminishing platelet activation (Schafer et al., 1980) and the anti-inflammatory effect of inhibiting leukocyte adhesion to the endothelium (Kubes et al., 1991). NO also leads to indirect vasodilation by inhibiting sympathetic vasoconstriction (Zanzinger et al., 1994). This is only a very small selection of a strong body of work that displays the role of NO in vascular function, which has been recently reviewed (Lundberg et al., 2015).

Since hemoglobin is present in the blood at a concentration of 10 mM in heme and ferrous heme has a strong affinity for NO with a dissociation constant of K_D_ 10^−10^ to 10^−11^ M (Cooper, 1999), and the association rate constant is 10^7^-10^8^ M^−1^s^−1^ (Cassoly and Gibson, 1975), you might expect there to be no free NO at all.

(1) $$\text{HbFe}(\text{II})+\text{NO }\overset{fast}{\underset{slow}{⇄}}\text{ HbFe}(\text{II})\text{NO}.$$

Moreover, oxygenated hemoglobin reacts with NO to form nitrate (dioxygenation of NO) extremely fast (6–8 × 10^8^ M^−1^s^−1^) (Doyle and Hoekstra, 1981; Eich et al., 1996; Herold et al., 2001) inactivating the NO, so that calculations predict that NO could not act as the EDRF (Lancaster, 1994; Vaughn et al., 1998).

(2) $$\text{HbFe}(\text{II}){\text{O}}_{2}+\text{NO}\to \text{HbFe}(\text{III}){+\text{NO}}_{3}^{-}.$$

However, as demonstrated above in the work by Chu et al. endothelial NO does play a physiological role in vascular function (Chu et al., 1991).

One can explain this paradox of NO bioavailability and Hb concentration in the blood by considering Hb encapsulation by RBCs. Experiments where RBCs are mixed with NO in suspension or in a stopped flow apparatus show that RBCs scavenge NO 1,000 times slower than cell-free Hb (Liu et al., 1998; Vaughn et al., 2000). Researchers have attributed this change in the rate of scavenging to a few mechanisms. One mechanism is the diffusion of NO to the RBC, which contributes to an unstirred layer around the cell (Coin and Olson, 1979; Liu et al., 1998, 2002; Azarov et al., 2005). A second mechanism is the membrane of the RBC acting as a barrier to diffusion (Vaughn et al., 2000; Huang et al., 2001, 2007). Third is diffusion of NO inside the RBC (Vaughn et al., 2000; Sakai et al., 2008). Our work suggests that rate limitations due to diffusion to the RBC are predominant (Azarov et al., 2005, 2011).

In addition to these changes under static conditions, a cell free zone created by the fluid dynamics of blood flow further reduces NO scavenging. The velocity of blood near the vessel wall is slower than in the center of the vessel due to friction. This creates a pressure gradient that pushes RBCs toward the center of the vessel away from the NO producing endothelium. Bugliarello and Sevilla, Cokelet and Liao et al. experimentally showed the presence of a cell free zone and computational modeling by Butler et al. supported its ability to reduce NO scavenging (Bugliarello and Sevilla, 1970; Cokelet, 1972; Butler et al., 1998; Liao et al., 1999). Subsequently, Liao et al experimentally demonstrated its ability to reduce NO scavenging using arteriole myography (Liao et al., 1999). Therefore, the compartmentalization of Hb inside the RBC and the localization of RBCs to the center of vessels have a strong influence on vascular function by minimizing NO scavenging.

## RBC disease—NO scavenging

In disease, RBC membrane abnormalities promote thrombosis (Ataga, 2009). Setty et al. showed exposure of phosphatidylserine on RBC membranes correlates with plasma indicators of thrombosis (Setty et al., 2001). In addition, changes to the RBC membrane lead to RBC adhesion to the endothelium. Diseases such as malaria, beta-thalassemia, diabetes mellitus, and sickle cell disease show increased RBC adhesion to endothelial cells (Kaul et al., 1989; Hovav et al., 1999; Cooke et al., 2004). Adhesion disrupts the cell free zone, which brings RBCs and Hb closer to the endothelium. Hoover et al. showed increased adhesion of RBCs from sickle cell patients to endothelial cells (Hoover et al., 1979) and Hebbel et al further demonstrated sickle RBC adhesion and suggested the adhesion may be a pathogenic factor in microvascular occlusion (Hebbel et al., 1980b). Hebbel also demonstrated a correlation between erythrocyte adhesion and sickle cell disease severity (Hebbel et al., 1980a). Kaul et al. developed a two-stage model of RBC adhesion initiating vaso-occlusion (Kaul et al., 1989). Thus, a role of RBC adhesion in initiating vaso-occlusive crisis is a widely supported hypothesis (Manwani and Frenette, 2013).

In addition to adhesion, polymerization of hemoglobin in sickle cell disease leads to rigid, inflexible red blood cells, which effect blood rheology. Moreover, oxygenated sickle RBC also displays changes in cell mechanics and therefore blood rheology, attributed to altered hemoglobin concentration in the cell and changes to the cell membrane (Nash et al., 1984). Changes in blood rheology affect tissue perfusion and may affect NO production through changes in shear stress on vessel walls. In addition to sickle cell disease, many other diseases display altered RBC deformability. In hypertension RBC deformability decreases, blood viscosity increases and RBCs form more stable aggregates (Cicco and Pirrelli, 1999). These changes exacerbate hypertension and effect oxygen transport (Cicco and Pirrelli, 1999). Diabetes and hypercholesterolemia, both risk factors for cardiovascular disease, show abnormal RBC membrane architecture and impaired deformability, which alter blood rheology (Shin et al., 2005; Tomaiuolo, 2014). It is difficult to determine if altered RBC deformability and therefore blood rheology are concurrent or contributing factors to the changes in vascular function in these diseases.

Work by Bor-Kucukatay et al. suggest vascular function, specifically nitric oxide production could play a role in RBC deformability and rheological alterations to RBCs during hypertension (Bor-Kucukatay et al., 2000, 2003). They suggest NO improves RBC deformability (Bor-Kucukatay et al., 2003). However, other labs have found that NO does not have a direct effect on RBC deformability (Barodka et al., 2014; Belanger et al., 2015). On the other hand, select NO donors, sodium nitroprusside and nitrite, protect against calcium-influx induced RBC dehydration and loss of deformability (Barodka et al., 2014; Belanger et al., 2015; Wajih et al., 2017). Use of sodium nitroprusside and nitrite to improve rheology in diseases affected by poor RBC deformability would thus be worth exploring.

As reviewed above, encapsulation of Hb inside RBCs greatly reduces its ability to scavenge NO produced by the endothelium. However, in multiple diseases RBC hemolysis occurs introducing cell-free Hb into the vasculature (Reiter et al., 2002; Sobolewski et al., 2005). The pathology associated with hemoglobin-based blood substitutes demonstrated the hypertensive effects of cell-free Hb (Doherty et al., 1998). Additionally, there is support for the implication of hemolysis in vaso-occlusive crisis (VOC) as hemolytic transfusion reactions can precipitate VOC (Jang et al., 2011; Manwani and Frenette, 2013).

Cell-free hemoglobin reactions in the vasculature and surrounding tissues through extravasation may add to disease conditions (Schaer et al., 2013). *In vitro* findings suggest cell-free hemoglobin has the potential to release hemin and take part in oxidative reactions resulting in oxidative stress and inflammation (Miller et al., 1997; Jia et al., 2007; Manwani and Frenette, 2013). Patients with elevated plasma Hb show a reduction in NO-dependent blood flow response, consistent with a decrease in vasodilatory response to NO donor sodium nitroprusside by patients with SCD (Rother et al., 2005).

In addition to releasing Hb, hemolysis also releases arginase, an enzyme that converts L-arginine to ornithine, into the blood stream. L-arginine is the substrate for nitric oxide synthesis by eNOS and therefore, the release of arginase further diminishes NO production and vascular function (Morris et al., 2003; Schnog et al., 2004). Hemolysis and changes to RBC membrane proteins in disease effect vascular function through promoting thrombosis, initiating vascular occlusion, scavenging NO, and oxidative stress.

Red blood cell breakdown not only decreases NO bioavailability through NO scavenging by cell-free Hb, but also by production of red blood cell microparticles (Donadee et al., 2011; Liu et al., 2013). Like cell-free Hb, red cell microparticles (on the order of 50-100 nm in diameter) scavenge NO hundreds of times faster than Hb encapsulated in the RBC, but not quite as fast as cell-free Hb (Donadee et al., 2011). In addition, these particles enter the cell-free zone (Liu et al., 2013). Red cell hemolysis and micropartcile formation have been proposed to contribute to poor outcomes associated with transfusion of older stored blood due to NO scavenging (Gladwin and Kim-Shapiro, 2009; Donadee et al., 2011). Substantial evidence suggests that extravasation plays a major role in NO dysregulation, so that proper compartmentalization of Hb is key (Kim-Shapiro and Patel, 2016; Schaer et al., 2016).

RBC hemolysis contributes to the vascular pathology of diseases and disorders such as thalassemia, hereditary spherocytosis, Glucose-6-phosphate dehydrogenase deficiency, paroxysmal nocturnal, hemoglobinuria, and autoimmune hemolytic anemia (Johnson et al., 1979; Rother et al., 2005). Additionally, these diseases and disorders also lead to the increased formation of RBC microparticles (Piccin et al., 2007; Westerman and Porter, 2016). Moreover, hemolysis is also associated with blood transfusion, hemodialysis and cardiac bypass surgery (Meyer et al., 2010).

## Production of nitric oxide

In contrast to the role played by RBCs in diminishing NO. Research shows deoxy-RBCs promote vasodilation in the presence of nitrite (Cosby et al., 2003; Jensen and Agnisola, 2005; Crawford et al., 2006). Other mechanisms of vasodilatory action by RBCs have been proposed. Researchers continue to debate the mechanism or mechanisms of hypoxic vasodilation which include: (1) ATP release by RBCs due to deoxygenation, (2) SNO-Hb formation and S-nitrosothiol release and delivery during oxy/deoxy hemoglobin cycling, and (3) nitrite reduction by hemoglobin to NO.

ATP activates purinoceptors on endothelial cells leading to the production of NO and alteration of vascular tone (Ralevic and Burnstock, 1991). In line with the mechanism of ATP release by RBC under hypoxia, Ellsworth et al. showed that RBCs release more ATP under low PO2 and low pH than RBCs under normoxia and normal pH (Ellsworth et al., 1995). Additionally, they demonstrated intraluminal ATP increased vessel diameter and flow rate (Ellsworth et al., 1995). Following this work, Dietrich et al. showed perfusion of RBCs at low PO_2_ caused vessel dilation and an increased concentration of ATP in the effluent (Dietrich et al., 2000).

Addition of nitrite to deoxyRBCs showed an enhanced effect on vasodilation when infused (Cosby et al., 2003; Jensen and Agnisola, 2005; Crawford et al., 2006). In support of ATP release being responsible for this enhanced dilation in the presence of nitrite, Cao et al. measured an increased synthesis and hypoxic release of RBC ATP in the presence of nitrite (Cao et al., 2009). However, research showed a vasodilatory response in the presence of nitrite and RBCs when NOS inhibitors L-NAME and L-NMMA were used, suggesting a different mechanism of dilation is responsible for the enhancement by nitrite (Crawford et al., 2006; Liu et al., 2015).

The second proposed mechanism for the effect deoxyRBC and nitrite on vascular function is the formation of SNO-Hb in the presence of nitrite and subsequent release of NO from deoxygenated SNO-Hb. Research has demonstrated the hypoxic release of NO from SNO-Hb and the ability of the released NO to relax vessels (Stamler et al., 1997; McMahon et al., 2002). Diesen et al. showed a decrease in RBC SNO-Hb concentration correlated with abolished vessel hypoxic dilation (Diesen et al., 2008). The proposed mechanism of release is a reduction in the stability of SNO-Hb when Hb transitions from the R-state to the T-state and work by Doctor et al. further supports the proposal coupling SNO-Hb concentration to oxygen saturation (Doctor et al., 2005). However, for this mechanism to be relevant *in vivo* SNO-Hb must form during the oxygenation/deoxygenation cycle of RBCs. This leads to the question of how SNO-Hb forms. Huang et al. and Xu et al. ruled out allosterically-controlled transfer of NO from HbNO to the beta-93 cysteine of Hb (Xu et al., 2003; Huang et al., 2006). In addition, the role of nitrite in the formation of a semi-stable metHb-NO intermediate capable of SNO-Hb formation was ruled out by Basu et al. (Angelo et al., 2006; Nagababu et al., 2006; Basu et al., 2007). Lastly, Isbell et al. demonstrated nitrite associated NO bioactivity in beta-93 knockout mice, demonstrating SNO-Hb is not responsible for nitrite associated hypoxic vasodilation (Isbell et al., 2007). While data supports the release of NO from SNO-Hb under deoxygenated conditions, research has yet to elucidate a physiological mechanism of SNO-Hb formation therefore bringing to question the physiological relevance of SNO-Hb in vascular function.

The last proposed mechanism of RBC vasodilation in the presence of nitrite is the reduction of nitrite to NO by Hb. Brooks first studied the nitrite Hb interaction in 1937 and this work was later extended by Doyle in 1981 (Brooks, 1937; Doyle et al., 1981). Nitrite reacts with deoxygenated Hb to form metHb and NO (Brooks, 1937; Doyle et al., 1981). NO then reacts rapidly with deoxygenated Hb, at a rate dependent on the conformation state of the Hb (Huang et al., 2005a,b), to form HbNO. *In vitro* studies have rejected a significant role in the reduction of nitrite by other RBC molecules such as xanthine oxidoreductase and carbonic anhydrase supporting the hypothesis that hemoglobin plays a dominant role (Liu et al., 2015). Due to the rapid scavenging of NO by Hb the question of how NO bioactivity escapes the RBC still remains. However, data continues to grow in support of NO bioactivity escaping the deoxy-RBC in the presence of nitrite. For example, platelets activation and aggregation by ADP is diminished in the presence of deoxyRBCs and nitrite (Srihirun et al., 2012; Park et al., 2014; Liu et al., 2015; Wajih et al., 2016). Platelets stimulated by ADP in the presence of nitrite alone do not show reduced activation (Srihirun et al., 2012). The addition of a NO scavenger to platelets stimulated by ADP in the presence of deoxy-RBCs and nitrite abrogates the diminished activation (Wajih et al., 2016). These data strongly support a role of RBCs in bioactivating nitrite.

However, once NO is formed inside a RBC, it must somehow escape rapid scavenging by the intraerythrocytic Hb. Proposed mechanisms of escape of NO bioactivity from the RBC have included S-nitrosothiol formation and the role of the RBC membrane. Research by Basu et al and Roche et al suggested S-nitrosothiol formation could occur through a metHb-nitrite mediated N_2_O_3_ formation (Basu et al., 2007; Roche et al., 2013). Pawloski proposed a mechanism where low molecular weight S-nitrosothiols could transnitrosylate AE1, an abundant RBC ion transporter, and eventually escape the RBC (Pawloski et al., 2001). Another proposed mechanism, is an increased affinity of metHb-NO or Hb-NO^+^ for the RBC membrane, which in turn could also lead to NO escape through nitrosylation of AE1 (Salgado et al., 2015). Lastly, Wajih et al have published evidence supporting a role of RBC membrane nitrosation in the escape of NO bioactivity (Wajih et al., 2016). While this is a start in determining the mechanism of NO bioactivity, much work remains.

Other heme-globin nitrite reducers may play a role in modulating vascular function. Deoxymyoglobin reduces nitrite faster than Hb (Shiva et al., 2007). Nitrite reduction by vascular smooth muscle myoglobin has been shown to contribute significantly to vasodilation in a murine model (Ormerod et al., 2010). Neuroglobin and cytoglobin also have nitrite reducing activity that is modulated by cysteine oxidation state (Tiso et al., 2011; Li et al., 2012; Tejero et al., 2014; Amdahl et al., 2017). Globin X from fish red blood cells reduces nitrite about 200 times faster than Hb and may represent a primordial function of heme-globins in nitrite reduction (Corti et al., 2016).

## Therapeutics for RBC associated vasculopathy

The production of NO by deoxyRBCs in the presence of nitrite and other NO donors provide a potential therapeutic role for plasma nitrite in reducing vasoconstriction, RBC adhesion, and potentially improving RBC deformability (Space et al., 2000; Bor-Kucukatay et al., 2003; Lundberg et al., 2008; Horn et al., 2011). NO administration can neutralize the NO scavenging ability of cell-free Hb by preferentially reacting, converting oxygenated hemoglobin to non-scavenging methemoglobin (Reiter et al., 2002). Antioxidants aim to improve blood rheology and vascular function through reduction of oxidative stress, shown by researchers to exacerbate hemolysis and endothelial dysfunction (Fibach and Rachmilewitz, 2008; den Hartog et al., 2010). Hemin and hemoglobin scavenger proteins also provide a potential therapeutic against damage caused by cell free hemoglobin and hemin (Schaer et al., 2013). Lastly, adhesion molecule inhibitors may act as a therapeutic against RBC adhesion to the endothelium; for example, Hebbel et al. showed adhesion molecule inhibitor HDAC reduced endothelial activation (Hebbel et al., 2009; Vinchi and Tolosano, 2013). A recent trial of a compound that interferes with adhesion of neutrophils and other circulating blood cells to endothelial cells successfully decreased the frequency of painful crises in patients with sickle cell disease (Ataga et al., 2017).

## Conclusions

Red blood cells play an important role in vascular function. Through the compartmentalization of Hb, RBCs deliver oxygen, minimize NO scavenging, sense oxygen tension, and deliver NO bioactivity in hypoxia. In healthy conditions, RBCs promote hemostasis through the well-regulated delivery of oxygen and the balance of NO scavenging and production. As data has shown, the RBC, once only viewed as a sink for NO, has the ability to produce NO bioactivity under hypoxia. The important role of the RBC in vascular function becomes more evident in diseases that alter or compromise the RBC membrane leading to conditions of oxidative stress, hypertension, thrombosis, and vaso-occlusion.

## Author contributions

CH, MG, and DK-S together conceptualized the article and its content. CH wrote the initial draft of the manuscript and further edited the manuscript. MG and DK-S heavily edited the manuscript.

### Conflict of interest statement

The authors declare that the research was conducted in the absence of any commercial or financial relationships that could be construed as a potential conflict of interest.
